# Noninvasive Abiotic Stress Phenotyping of Vascular Plant in Each Vegetative Organ View

**DOI:** 10.34133/plantphenomics.0180

**Published:** 2024-05-22

**Authors:** Libin Wu, Han Shao, Jiayi Li, Chen Chen, Nana Hu, Biyun Yang, Haiyong Weng, Lirong Xiang, Dapeng Ye

**Affiliations:** ^1^ College of Mechanical and Electrical Engineering, Fujian Agriculture and Forestry University, Fuzhou 350002, China.; ^2^ Fujian Key Laboratory of Agricultural Information Sensing Technology, College of Mechanical and Electrical Engineering, Fujian Agriculture and Forestry University, Fuzhou, Fujian 350002, China.; ^3^ Center for Artificial Intelligence in Agriculture, School of Future Technology, Fujian Agriculture and Forestry University, Fuzhou 350002, China.; ^4^ Department of Biological and Agricultural Engineering, North Carolina State University, Raleigh, NC 27606, USA.

## Abstract

The last decades have witnessed a rapid development of noninvasive plant phenotyping, capable of detecting plant stress scale levels from the subcellular to the whole population scale. However, even with such a broad range, most phenotyping objects are often just concerned with leaves. This review offers a unique perspective of noninvasive plant stress phenotyping from a multi-organ view. First, plant sensing and responding to abiotic stress from the diverse vegetative organs (leaves, stems, and roots) and the interplays between these vital components are analyzed. Then, the corresponding noninvasive optical phenotyping techniques are also provided, which can prompt the practical implementation of appropriate noninvasive phenotyping techniques for each organ. Furthermore, we explore methods for analyzing compound stress situations, as field conditions frequently encompass multiple abiotic stressors. Thus, our work goes beyond the conventional approach of focusing solely on individual plant organs. The novel insights of the multi-organ, noninvasive phenotyping study provide a reference for testing hypotheses concerning the intricate dynamics of plant stress responses, as well as the potential interactive effects among various stressors.

## Introduction

Plant response to stress is a dynamic equilibrium process, if attainable, accompanied by physiological and morphological changes in different organs. The major goal of these adjustments is to reach a new balance [1]. Therefore, the detection of plant stress is crucial for optimal plant growth and development, particularly in light of the increasing global population and the growing threat of extreme weather events [2–4]. Plant stress means that the sub-healthy state caused by stress factors, which, if exceeded tolerance, can cause permanent damage [1,5]. Unlike animals that can move away to avoid adverse environments, plants have to remain there and face the challenges. Plants have evolved a multitude of strategies to survive or even thrive through environmental/abiotic challenges, including cell metabolism and physiological and morphological changes. This is also why plants exhibit phenotypic plasticity [6], which refers to their ability to alter their phenotypic form in response to stress, and these are stress phenotypes we aim to obtain.

To obtain thorough phenotypes while minimizing interference, noninvasive phenotyping is widely used, without requiring physical splitting or biochemical extraction as traditional invasive methods do. Noninvasive phenotyping also has the distinct advantage of being comparable and reproducible, with the potential to realize kinetic monitoring of the growth and development of the same organs without obvious and serious disturbance [7–9]. Noninvasive phenotyping can be achieved through optical sensing, utilizing optical waves (the light wave and type of electromagnetic radiation) to interact with the object and feedback spectral characteristics [10,11]. Optical methods vary from nonimaging spectroscopy to imaging methods, such as visible [12], NIR (near-infrared) [13,14], multispectral [15,16], hyperspectral [17,18], thermal-IR [19], and chlorophyll fluorescence (ChlF) methods [20,21], as well as computer tomographic (CT) [22,23], light detection and ranging (Lidar) [24,25], magnetic resonance imaging (MRI) [26], and positron emission tomography (PET) [27] techniques. All have been intensively studied to acquire data for quantitative studies of characteristics related to vascular plant stress [28–31]. Vascular plants, once known as higher plants (no longer used because it is not accurate enough), form a large group of land plants (approximately 374,000 accepted plant species, of which approximately 308,312 are vascular plants) [32,33]. As the name implies, vascular plants have vascular tissues, which are composed of xylem (for transporting water and minerals throughout the plant) and phloem (for conducting products of photosynthesis). The xylem and phloem are typically located adjacent to each other and form vascular bundles, acting as a transport system in the plant [33]. Typically, vascular plants possess vegetative organs (leaf, root, and stem) and reproductive organs (flower, fruit, and seed). Here, we target vegetative organs, as they are closely linked to plant stress resistance, while reproductive organs are more related to propagating offspring.

Root, stem, and leaf are homologous structures in vascular plants, meaning they share similarities in structure and evolutionary origin, although their functions may differ [34]. The root anchors the plant in the soil and absorbs water and minerals. The leaf is the main photosynthetic organ, while the stem acts as the connecting and communication channels between organs. Conventionally, when it comes to determining which organ fits better to monitor stress, most are just done on leaves. Plant leaves certainly would reflect meaningful information but may not be enough. The reasons are as follows: (a) the leaves of one plant in different positions or stages would present considerable differences; (b) even if all leaves changed consistently, it is difficult to distinguish what stress causes the change, as diverse stress often leads to similar tangible changes in leaves [1,35]. Therefore, to make the distinction, it is necessary to consider stress indicators of other organs comprehensively. Hence, in this research, each vegetative organ view under corresponding abiotic stressors is analyzed, emphasizing the importance of distinguishing the phenotypic information provided by each organ. The review article can be divided into 7 parts. The “Introduction” section is the introduction part. The “Abiotic Stress and Noninvasive Phenotyping Overview” section provides a comprehensive overview of plant stress and phenotyping methods. The “Leaf View under Abiotic Stress”, “Stem and Whole Plant View under Abiotic Stress”, and “Root View under Abiotic Stress” sections present a detailed discussion of how each vegetative organ perceives and responds to various environmental stress, as well as their phenotyping technologies. The “Compound Abiotic Stress Phenotyping” section explores the probability of analyzing complex or compound abiotic stress, namely, 2 or more stressors worked simultaneously or subsequently, as one single organ view would sometimes lead to confusion. Finally, the “Conclusions and Perspectives” section summarizes the advantages and challenges of different vegetative organs' view in stress phenotyping.

## Abiotic Stress and Noninvasive Phenotyping Overview

**Fig. 1.**
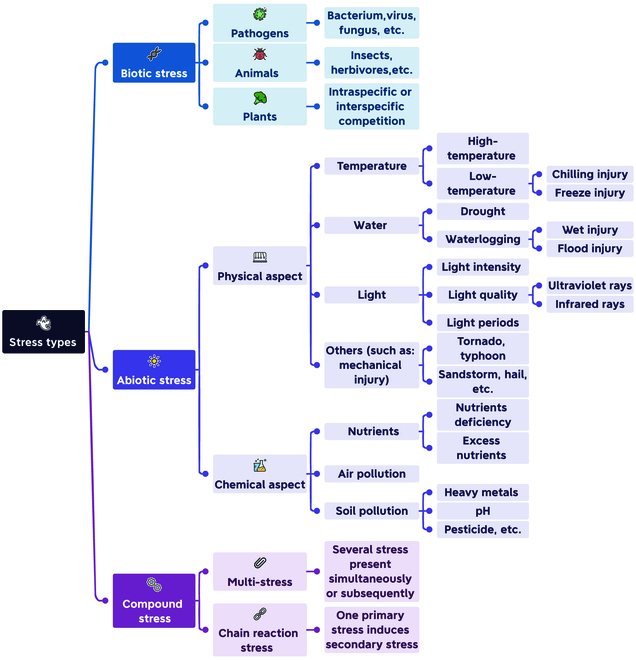
An overview of stress types: biotic, abiotic, and compound stress.

Fluctuations are the nature of the environment [36]. Either natural factors (such as circadian rhythm, seasonal change, and weather variability) or artificial interference (such as chemical pollution and physical radiation) would affect the plant’s homeostasis. Being sessile, plants have evolved strategies that allow them to maintain steady internal conditions, while higher doses and/or longer duration can lead to severe stress. There are various stress types, and Fig. 1 shows a comparatively detailed classification.

Abiotic stress means non-biological stress, which can be categorized into physical and chemical aspects. Compound stress includes multiple stresses; different types of stress happen simultaneously or subsequently; and chain reaction stress and secondary or tertiary stress are caused by primary stress. Biotic stress means biological stress, which can be further categorized into pathogen, animal, and plant stress. As various types of living organisms can attack plants, ranging from macro- to microorganisms, affecting leaves to roots, and eliciting diverse responses, for the sake of simplicity and clarity, our primary focus in this study is on abiotic stress and compound stress.

**Fig. 2.**
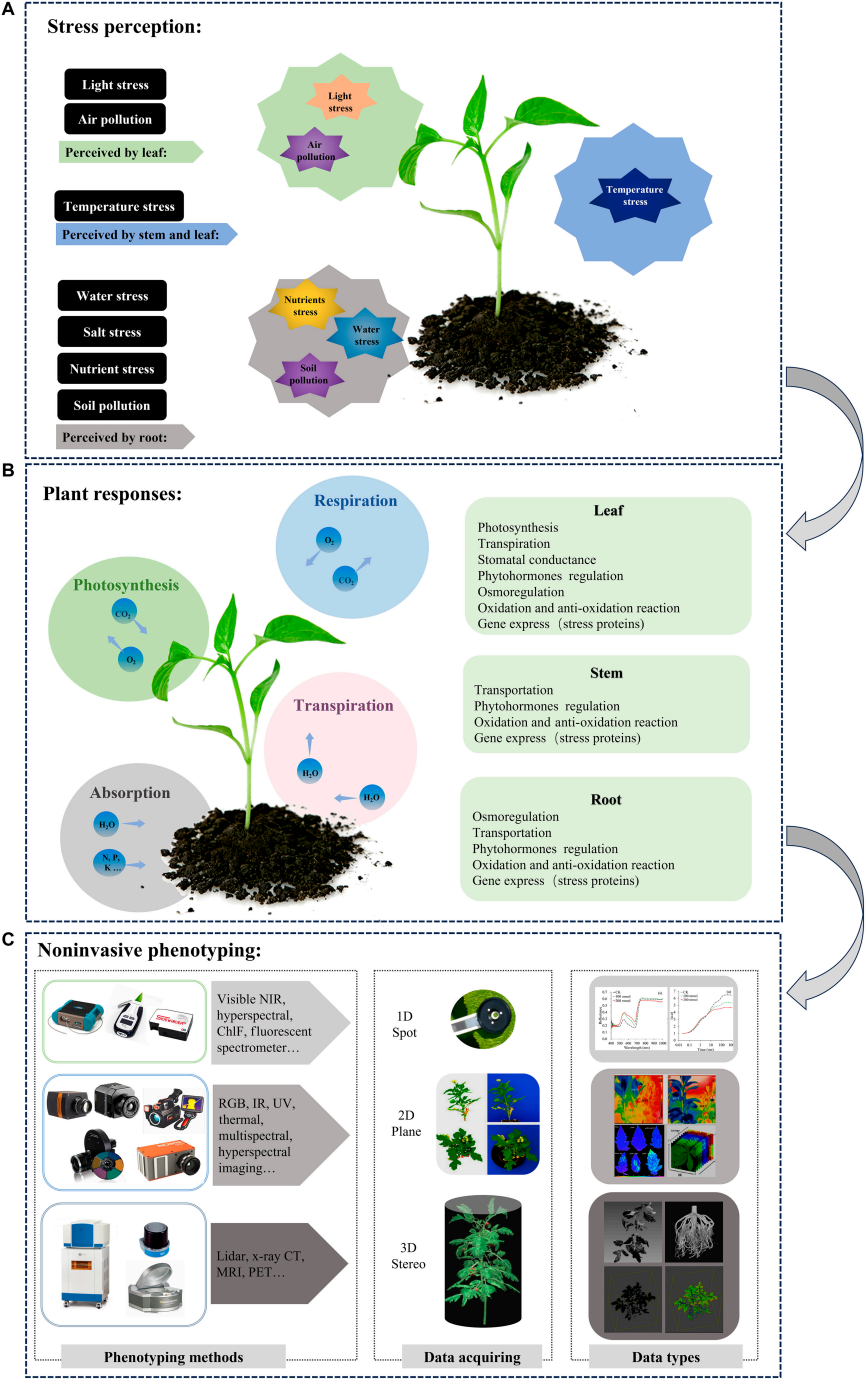
Noninvasive abiotic stress phenotyping: from stress perception to phenotyping techniques. (A) Stress perception from each vegetative organ view. (B) Plant responses to abiotic stress from each vegetative organ view. (C) Noninvasive phenotyping techniques in various dimensions, including 1-dimensional (1D) spot phenotyping, 2D imaging, and 3D stereo phenotyping. Abbreviations: RGB, red–green–blue imaging; IR, infrared imaging; UV, ultraviolet; CT, computed tomography; MRI, magnetic resonance imaging; PET, positron emission tomography.

Despite the multitude of abiotic stressors, when considering just vegetative organs, they are perceived solely through 3 organs: leaf, stem, and root. Each vegetative organ is an integral part of one plant system, determining its survival and development. According to the location of the stressors, whether in the air or under the soil, the plant senses them through the corresponding organs. Subsequently, the plant initiates specific physiological, biochemical, molecular, and morphological adjustments to cope with these stressors.[37]. Leaves exposed to the air perceive air pollution first. Roots growing underground perceive drought stress or salt-alkaline stress first, while stem and branches, relating considerably to plants’ water and nutrient transportation, are primarily associated with low-temperature freezing stress [38]. The leaves synthesize sugars and release O_2_, the roots absorb water and dissolved minerals from the soil, and the stem connects leaves and roots [39]. That is to say, the responses of different vegetative organs under stress should be considered separately at first and then assessed comprehensively. Subsequently, this information can be leveraged to implement corresponding noninvasive phenotyping methods. These techniques encompass 1-dimensional (1D) spot phenotyping, 2D imaging, and 3D stereo phenotyping, which collectively enable a comprehensive evaluation of plant stress [40], as Fig. 2 shows.

Hence, the discussion that follows introduces various abiotic stressors in leaf view (light stress and air pollution), stem or whole plant view (temperature stress), and root view (soil pollution and water stress). Here, the stem and whole plant view are categorized as one part for the reason that stem connects the whole plant, and its central function part is the vascular tissue [33], which is responsible for the conduction of water and nutrients throughout the plant.

## Leaf View under Abiotic Stress

Leaf possesses 2 crucial physiological functions: photosynthesis (interacting with light and producing sugar and O_2_) and transpiration (water evaporation**,** supporting power for water, and nutrient transportation) [39]. Photosynthesis is a series of energy and chemical transformation processes that mainly take place within organelles called chloroplasts, while transpiration is primarily controlled by guard cells, which are further determined by light or water potential [41]. Photosynthesis and transpiration are vital for one plant’s surviving and thriving. Being exposed to the atmosphere, leaves perceive light stress and atmosphere pollution, thus affecting their physiological function and spreading to other organs, as described below.

### Light stress

Light, especially sunlight, is essential for plant photosynthesis and growth development. Due to fluctuating natural conditions (cloudy shading or overlapped by other leaves), light may be the most common stress [42]. Notably, even in a greenhouse, an artificial light source can hardly match the full spectrum of solar radiation and thus may cause light quality stress.

The most obvious manifestation of light stress is photosynthesis. Both insufficient and excess lighting would limit the photosynthetic rate. Under insufficient light conditions, chloroplasts capture fewer photos and produce less NADPH and ATP; thus, they cannot realize their optimal photosynthesis ability. However, under excess light intensity, as chlorophyll has a capacity for transporting electrons to produce products like NADPH and ATP, those extra electrons would overflow and lead to excessive generation of oxygen radicals and reactive oxygen species (ROS), which will damage chloroplast components [43,44]. Moreover, excess light may also lead to high-temperature stress, thus causing secondary damage. There also exists light quality stress [45]. Those invisible lights, such as UV (200 to 380 nm) and IR (780 to 2,500 nm), may not be directly involved in photosynthesis but are also necessary for plant development [45,46]. In summary, light stresses would ultimately induce photoinhibition, leading to excessive ROS generation. Excess ROS would damage organelles, proteins, membrane lipids, and cell vigor and subsequently affect other physiological activities, and these changes could manifest themselves in the leaf's spectral characteristics [47,48].

### Atmosphere pollution stress

Plants exchange gases and water vapor with the atmosphere all the time, mainly through the stomata (as shown in Fig. 2, formed by 2 guard cells located in the backside leaf's epidermis) [39]. Thus, when exposed to polluted air, leaves perceive pollutants. Air pollutants include fine suspended particles (e.g., PM_10_, PM_2.5_), gas pollutants (NO, SO_2_, NOx, O_3_, and N_2_O), photochemical smog, volatile organic compounds [49], etc. All of them can cause damage to leaf tissue’s vigor. That is also why leaves are widely used as a system for monitoring air pollution [50]. Leaves adsorb and accumulate air pollutants, if overdosed, which would induce harmful morphological and biochemical changes [51,52], such as damaging foliar tissue, destroying protein structure, affecting metabolic processes, and resulting in a series of metabolic disorders [50,53,54].

Air pollution can affect the leaf’s main physiological functions, photosynthesis and transpiration. Photosynthesis is a well-explored subject encompassing various wavebands. Meanwhile, the leaf’s transpiration function can also respond to stress phenotypes, such as the research about gas stoma phenotyping [55]; the authors found that analysis of stomata density and its configuration based on the scanning electron microscopic image of a leaf surface is an effective way to characterize the plant’s behavior under various environmental stresses. Air pollution can also cause the deposition of contaminants in the soil, thus causing soil pollution, which would contaminate the environment of the rhizosphere system [56]. The studies conducted by Sanaeifar et al. [55,57] introduced the phenotyping of Pb pollution stress in tea plants, considering both leaf view air pollution and root view soil pollution. The authors researched tea seedlings under lead-containing aerosol particles stress and studied the effects of airborne Pb pollution on quality indicators and accumulation in tea plants using Vis (visible)–NIR spectroscopy. These studies not only represent classical examinations of air pollution through the leaf view but also provide a basis for further exploration into the underlying mechanisms of multi-organ view phenotyping.

### Leaf view phenotyping techniques

Under stress, the leaf can reflect not only external morphological traits (such as leaf color, size, and number) but also internal physiological properties (such as photosynthesis and transpiration). All these would form the leaf’s spectral or optical transmission characteristics, which have been intensively studied using optical sensing technologies, as listed in Table 1. Specifically, when light waves hit leaves, they are absorbed, transmitted, or reflected, but some even emit light after being struck, which is also known as fluorescence [58]. Considering this, general leaf view optical sensing technologies can be classified into Vis–NIR, multispectral, hyperspectral, thermal-IR, and ChlF spectroscopic or imaging methods [11,59]. Changes in spectral information can be associated with deleterious effects on the physiological and biochemical processes of plants, as demonstrated in the study by Feng et al. [60]. They revealed correlation coefficients among sodium concentration, photosynthetic rate, and transpiration rate, suggesting a meaningful relationship between these parameters and a fundamental regulatory mechanism in plants. Besides macroscopic optical methods, microscopic phenotyping is an important supplement for exploring microtissue or cellular structures, as well as organelle functions, to elucidate the physiological, biochemical, and molecular mechanisms governing plant responses to stressors. For example, the study conducted by Feng et al. [61] introduced the Organelle Segmentation Network in electron microscopy. This network enables pixel-wise segmentation; identifies chloroplasts, mitochondria, nuclei, and vacuoles; and provides valuable insights for effective microscopic plant phenotyping.

**Table 1.** Noninvasive stress phenotyping techniques in leaf view

Noninvasive phenotyping	Description	Measured traits (leaf view)	Pros/Cons	Reference
Visible waveband	Visible region (400–780 nm) can provide information on morphological properties and the content of pigments (such as chlorophyll a and b, carotenoid, and phytochrome).	Leaf size, projected area, color, number, canopy cover, canopy color, pigment distribution, green indices, and Red Edge indices.	Pros: Simplicity, accessible, portable	[29,123–125]
			Cons: Spectral information is limited in visual spectral bands	
NIR, SWIR	NIR (800–1,300 nm) and SWIR (1,300–2,500 nm) are associated with the measurement of overtones and combination tones of molecular vibrations (such as the C-O, C-H, O-H, and N-H covalent bonds of macromolecules).	Water content, nitrogen protein, cellulose, phosphorus, hemicellulose, protein, mineral contents, etc.	Pros: Suitable for screening multi-traits under stress conditions	[13,126];
			Cons: Vulnerable to meteorological conditions and needing background correction	
Multispectral/hyperspectral spectra	Hyperspectral (covering 250–2,500 nm, with nearly 0.1 to 1 nm resolution) contains ultraviolet, visible, NIR, and SWIR wavebands. Multispectral sensing is similar to hyperspectral sensing but with sparse wavelength information.	Various vegetarian indices, spectral reflectance indices, leaf water potential, biochemical composition, pigments concentration, water content, chlorophyll content, canopy architecture, etc.	Pros: A wide range of testing objects; with abundant spectral information, various vegetarian indices can be calculated to characterize sample features	[127–130]
			Cons: Data processing capacity; trade-offs in resolution, price, performance, and portability	
Thermal-IR	Thermal-IR imaging allows the visualization of temperature differences in the surface of plants caused by stress.	Stomatal conductance, canopy or leaf temperature, water content, etc.	Pros: Suitable for screening multi-traits under stress	[131,132]
			Cons: Need soil background correction	
Chlorophyll fluorescent	ChlF optical phenotyping is typically linked to active lighting, where leaves are excited by UV radiation or natural light, causing chlorophyll to emit fluorescent light. This emitted light is then recorded to assess leaf photosynthetic abilities.	Leaf health status, photosynthetic status, non-photochemical quenching, quantum yield, etc.	Pros: Providing a quick way to probe plant photosynthesis ability and parameters related to early stress	[58,133–135]
			Cons: Only leaf information is collected; photosynthesis parameters are vulnerable to various conditions and sometimes need dark adaptation	

NIR, near-infrared; SWIR, short infrared; ChlF, chlorophyll fluorescence.

## Stem and Whole Plant View under Abiotic Stress

Stem acts as the interaction and communication channel between organs, transporting water and substances throughout the plant, just like human blood vascular tissue. The stem’s vascular tissue is mainly composed of the xylem and phloem. The ascent of sap within xylem tissue, also termed sap flow, can be measured to illustrate the transpiration status and water usage [62]. They are also critical indicators for understanding the strategies and actions plants adapted for stress resistance. It should be noted that temperature stress can affect not only the stem but also other organs. Thus, it seems not rigorous enough to regard temperature stress only in the stem view. The case depends either on the temperature degree or on plants per se to view temperature stress in the stem or whole plant view. However, all would affect the sap flow rate and solutes in vascular tissue, which is distributed in the whole plant but the stem occupies the main part [63,64]. Moreover, freezing injury stress is most related to the stem [65], which is why we temporarily categorize temperature stress mainly in the stem view.

### Temperature stress

Temperature causes plant stress via 2 extremes: high-temperature stress and low-temperature stress. Low temperatures can be further divided into chilling injury (above 0°C, yet lower than the optimal temperature) and freezing injury (below 0°C, can freeze water in the plant) [66]. Extreme temperature can affect enzyme activity and thus affect a broad spectrum of physiological activity. Metabolism, a prerequisite to support life, is mainly catalyzed by enzymes, yet the enzymes are temperature-dependent [67]. Disrupting metabolism leads to the accumulation of toxic intermediates such as ROS, and causes damage to cell vigor, and further affected a series of physiological functions.

#### High-temperature stress

Extremely high temperature affects different organs’ vitality, limits metabolism, and slows growth, and further prolongation can lead to permanent damage or even death [68,69]. Moreover, high-temperature stress promotes water evaporation if it exceeds water uptaken, thus causing stress like drought, but more harmful for high-temperature hurt [70]. Stress-tolerant plants employ both morphological and physiological changes and molecular responses to alleviate high-temperature stress, for example, stimulating corresponding gene expression and accumulating stress-tolerance proteins [66,69,71]. These mechanisms could be varied due to plant cultivars.

#### Low-temperature stress

At low temperatures, such as chilling stress (0 to 15 °C), enzymic activity will be affected, and then the enzyme-dependent physiological and metabolic process will be limited [72]. Phenotypic symptoms in response to low-temperature stress included wilting and yellowing of leaves, stunted seedlings, and limited growth and development of the plant. Chilling-resistant plants tend to have a higher proportion of unsaturated fatty acids in their membrane that solidify slower than those containing more saturated fatty acids [72,73].

When the temperature drops below 0 °C, ice formation starts, causing freezing stress. The freezing point of water is related to solution concentrations. Thus, intercellular fluids freeze before the intracellular fluids due to lower concentration [74]. Ice formation in the intercellular fluids reduces the water potential, and then unfrozen water within the intracellular moves out. In this respect, freezing stress also causes dehydration stress or drought stress [75]. During this process, the membrane, rigidified by the low temperature, may lose elasticity and be unable to contract. The colder the temperatures, the higher the mechanical strain on the cell membrane, and the more dangerous the situation is for the plant [73]. Low-temperature stress is perceived by the receptor at the cell membrane, then switches on the expression of various cold-resistance genes [73]. For example, freezing-resistant plants can produce antifreeze proteins to limit the formation of ice crystals, thus improving freezing tolerance [71,76].

### Stem view phenotyping techniques

Stem acts as the delivery system of the plant, but few studies about stress phenotyping are concerned with the stem. Most of them are about morphological traits, such as height, diameter, and so on. Considering various stem structural, positional, and stage differences, the stem can be divided into the main trunk and branches, or it can be divided into fresh and old parts over time. However, changes in xylem and phloem functions under stress may share common mechanisms. Early experiments with dyes have widely been conducted to test stem sap flow, in the trunk, branches, or tillers [77]. Methods based on the heat dissipated by the ascending sap have also been studied, involving measurements of temperature changes around the heater or the time required for temperature transport [78,79]. Yet, studying these changes in xylem or phloem under stress conditions can be destructive and potentially harm the plant. Therefore, conducting non-destructive and high-throughput measurements of sap flow remains a challenging task.

Acoustic methods can be non-destructive [80–82], with the hypothesis that larger conduits produced lower frequency signals and smaller units emit the ultrasonic frequencies, which can be associated with stress response in plants. Meanwhile, electronic methods, such as stem sap flow sensors, are also feasible [83,84]. These sensors, being soft, thin, and wearable, enable continuous detection of stem transport, and the results obtained can serve as important cues for plant stress identification or prediction [83]. However, to refocus specifically on optical phenotyping, advanced high-resolution imaging methods, such as MRI, PET, and x-ray CT imaging, are promising ways to provide dynamic information on transport flows in the vascular system in response to stress [22,85,86]. These phenotyping techniques are listed in Table 2. The next major step will be developing portable, practical, and accessible devices to measure sap flow under real-field conditions.

**Table 2.** Noninvasive phenotyping techniques in the stem or whole plant view

Noninvasive phenotyping	Description	Measured traits (stem view)	Pros/Cons	Reference
Visible imaging	Visible imaging captures visible light and records the images on sensitive material.	Plant structure, branching angles, internode lengths, height, stem or branch diameter, etc.	Pros: Simplicity, portable, and accessible	[136,137]
			Cons: Limited to visual spectral bands information	
Wearable electronic sensor	The sensor can be made ultrathin, flexible, and wearable, thus can softly attach to the epidermis and provide continuous monitoring.	Stem sap flow, temperature and humidity, growth of stem or other organs, etc.	Pros: In situ monitoring, realize stem transport detection in a continuous and noninvasive manner	[83,138]
			Cons: Limited application species and measurement parameters	
Lidar	Lidar uses pulsed lasers to build point clouds to describe the 3D surface structure.	Plant architecture; LAI (leaf area indices); volume and biomass, etc.	Pros: Providing 3D architecture, capable of realizing high throughput	[25,139–141].
			Cons: Limited to laser light spectral, only provide surface and architecture information	
X-ray CT	X-ray CT is based on the attenuation of x-rays to create cross-section images.	Morpho-anatomical stem properties, stem length, diameter, and pithiness ratio	Pros: Collect both morphological and anatomical stem properties	[142,143]
			Cons: Time required, pay attention to safety issues	
MRI	MRI is based on the magnetic momentum nucleus (^1^ H, ^13^ C, etc.) using strong magnetic fields and radio frequency to differentiate their content and generate images of the internal structure.	Anatomical and structural traits, water use, certain metabolites, etc.	Pros: 3D noninvasive internal architecture; relatively high spatial resolution (up to 30 μm^3^ per voxel)	[144–146].[147]
			Cons: Homogeneous magnetic field, low throughput, bulkiness, and non-portable	
PET	PET is based on the detection of γ-rays from tracer molecules, thus can provide internal functioning information.	Water transport, sugar transport, flow velocity, dynamic interactions in the vascular tissues, etc.	Pros: 3D images, noninvasive internal architecture	[86,148] [27,149]
			Cons: Low throughput, high cost, limited resolution, restricted to short-term qualitative analyses	
MRI-PET	The combination of MRI and PET can obtain complementary information, providing a novel functional and structural imaging procedure.	Plant structures, vascular tissue transportation, transport routes, translocation dynamics, etc.	Pros: Providing detailed structural and functional information	[118]
			Cons: Technical compatibility**,** non-portable, larger 3D datasets requiring complex graphical representation	

Lidar, light detection and ranging; X-ray CT, x-ray computed tomography; MRI, magnetic resonance imaging; PET, positron emission tomography.

## Root View under Abiotic Stress

Root anchors the plant in the soil and absorbs water and other substances. The process of water and dissolved nutrient absorption by roots is shown in Fig. 2. Most water and dissolved minerals in the soil are absorbed by the root hairs, which are permeable and hydrophilic. A large number of root hairs also substantially increase the surface area of the root, thus providing greater capacity for the effective absorption of water and nutrients [87]. The movement of water and other substances from the soil into the root requires osmosis to work collaboratively [39]. These crucial substances then make their way up the plant to other organs through the vascular tissues. Thus, roots first perceive water and mineral nutrient stress (water deficit, waterlogging, mineral nutrient deficiency, and nutrient excess) and soil pollution (heavy metal and other contaminants), which are further described as follows.

### Water stress

Water that is deficiently (drought) or excessively (flooding) supplied means stress to plants [88,89]. One can quickly tell if plants are under extreme drought stress or flooding stress. However, when it comes to subtle conditions, like under soil or hydroponic cultivation, the identification must be done through fine phenotyping.

#### Water deficit stress

Plants need to balance the absorption and evaluation of water all the time, which is vital for the plant’s transportation cycle. Root water absorption that fails to keep up with leaf evaluation induces water deficit or drought stress [39]. Of all the resources, the water deficit is the most severe factor threatening crop yields [90]. The main result of drought stress is dehydration. Typically, the apparent symptoms of water deficit stress are aboveground morphological phenotype, curling and wilting of leaves, and drooping of the plant's branches [88]. Then comes leaf stomatal closure [91], which can not only reduce transpiration and diminish water loss but also limit CO_2_ absorption, later followed by the alteration of chlorophyll content and the reduction of plant LAI (leaf area index) [92]. These all lead to a negative influence on the metabolic and osmotic balance [30]. Under drought stress, osmotic adjustment (OA) has been implicated in maintaining water content by increasing the accumulation of solutes to maintain turgor and promoting the growth of roots to increase water uptake capacity [93]. The sensitivity of plants to drought stress varies with species at different stages, and the most susceptible yet critical period for the crop is called the critical water period [92,94,95], which should be diagnosed and irrigated in time to avoid loss.

#### Water logging stress

Water logging means excessive amounts of water in the soil around the roots, which could reduce gas exchange and result in hypoxia or anoxia stress. Both hypoxia and anoxia describe stress conditions in that plants receive insufficient oxygen, also known as anaerobic stress [96]. Gases, like oxygen, can be dissolved in the soil water solution, but roots primarily exchange gases through the air-filled pores between soil particles. Thus, waterlogging stress leads to limited oxygen and other nutrient absorption. Waterlogging changes their energy metabolism, such as respiration [97]. Aerobic respiration (oxygen-requiring) is suppressed, and anaerobic respiration (does not require oxygen) is enhanced. This metabolic shift can cause accumulation of alcohol, acidification of the cytosol, and toxicity to the root cells, and hamper root absorption [98,99]. Such reduction will result in decreased nutrient uptake, cell maintenance, and plant growth [89,98]. In this way, too much water leads to drought stress instead [89].

### Mineral nutrient stress

Chemical analysis revealed 17 elements that are essential for plant growth and metabolism. Except for C, H, and O, which mainly come from H_2_O and CO_2_ in the air, another 14 elements are primarily absorbed from the soil [100]. Although some evidence shows that plants can absorb nutrients and water through foliage [101], this method is limited and does not fit all nutrients. Mineral stress can be caused either by high concentrations or low availability of these elements.

#### Mineral nutrient excess stress

Adding excess nutrients causes osmotic stress to the plant. Extra nutrients and mineral ions in the soil may inhibit water and minerals absorption and limit plant growth, just like drought stress, but more harmful than high-density ion toxicity injury. The presence of excess levels of the particular mineral nutrient can also influence the pH of the soil solution, thus affecting the rhizosphere system. Furthermore, the excess supply of a particular mineral nutrient will induce a deficiency of other nutrients within the plant, resulting in detrimental effects on the plant [102,103]. The most common mineral nutrient stress is salt-alkaline stress, which widely happens in arid and semiarid regions, as rainfall is inadequate to leach too many minerals’ nutrients from the soil layers near the surface, and the soil is prone to be saline [99]. High amounts of salt taken up by a plant can lead to severe osmotic and ionic stress in plants. The former can cause plant hypoxia to lower water potential, disturb mineral uptake and transportation, and hamper photosynthesis, while the latter results in ionic imbalance, damages plant cells, distorts metabolic activity, and generates excess ROS content [104]. To avoid the accumulation of mineral ion toxicity, various resistance strategies have been taken by plants, including biochemical synthesis, enzyme induction, and membrane transport.

#### Mineral nutrient deficiency stress

Among the 14 elements that plant requires, N (nitrogen), K (potassium), Ca (calcium), Mg (magnesium), P (phosphorus), and S (sulfur) are in relatively large amounts (more than 0.1% of dry mass), termed macronutrients [105]. All are necessary to pursue sustainable yield, enhanced quality, and stress tolerance. Typically, the elements are obtained from soil, with the metals Ca^2+^, Mg^2+^, and K^+^ as free cations, and P, S, and N as their oxyanions (PO_4_^3−^, SO_4_^2−^, NO_3_^−^, or NH_4_^+^, respectively) [105]. Plants have highly sophisticated absorption mechanisms to adapt to fluctuations in soil nutrients. Between pH ranges of 5.5 and 6.5, the majority of mineral nutrients are accessible. However, if pH exceeds this range, most nutrients become insoluble, making them unavailable for absorption [106], thus also causing nutrient deficiency. As mineral nutrients are components of essential proteins and building blocks of the cell, deficiencies in one or more essential mineral nutrients would cause a wide range of disorders, but what they have in common is the suppression of plant growth and reproduction [102,103, 107]. An insufficient supply of nitrogen will result in certain morphological traits such as decreased leaf area, foliage discoloration, and plant dwarf, or physiological characteristics such as weakened photosynthesis, respiration, and other activities. Under nutrient deficiency conditions, different transcription factors and regulatory gene networks function together to maintain mineral homeostasis [108,109]. For more detail on the nutrient deficiency stress response, see [100].

### Soil pollution stress

Soil pollution, along with water pollution, means the presence of excess toxic chemicals, contaminants, or heavy metals in the rhizosphere, which would adversely affect plant root absorption. In addition, soil pollution has harmful effects on soil microorganisms, resulting in a change in the diversity, population size, and overall activity of the rhizosphere ecological system [110]. Take heavy metal (Cd, Pb, Ni, Cr, etc.) stress as an example. The uptake of heavy metals by plants can lead to subsequent accumulation along the food chain, which has become a serious concern [111]. A high content of heavy metals in plants can lead to essential physiological and biochemical complications, including inhibition of metabolism and enzymatic reactions, disruption of membrane structure and ion homeostasis, and activation of programmed cell death [112]. Heavy metals can mimic other essential metals, take their place in basic reactions, and disrupt them. Cd, for instance, can replace Mg in chlorophyll or Ca in the signaling protein, disrupting both photosynthesis and signal transduction [113,114].

### Root view phenotyping techniques

Noninvasive underground root phenotyping is more challenging than leaf and stem, as the soil is opaque to normal optical sensing (visible, multispectral, hyperspectral, and thermal-IR) methods. Applying transparent mediums (gel medium, hydroponics, and aeroponics) can solve this problem, but with doubts that it is inconsistent with normal field conditions [115]. To date, noninvasive 3D phenotyping underground in the field can be realized through x-ray CT, MRI, and/or PET techniques, the radiation of which can penetrate through the soil to obtain root architecture or even root internal structure [116,117]. However, they often need a relatively long time to form imaging (there are trade-offs in costs, scan time, reconstruction time, and resolution) and are often vulnerable to soil moisture content [118,119]. Thus, more advanced approaches are still needed to improve the root view phenotyping system and make it more portable and accessible. These noninvasive phenotyping methods in root view are shown in Table 3.

**Table 3.** Noninvasive phenotyping techniques in the root view

Noninvasive phenotyping	Description	Measured traits (root view)	Pros/Cons	Reference
Rhizotron (2D imaging)	Rhizotron is a growth chamber with transparent or removable observation windows through which roots can be imaged.	Root system architecture, root development, etc.	Pros: Observe root growth noninvasively	[150]
			Cons: 2D images would lose some architectural information	
ERT	ERT is based on the variation of soil electrical conductivity in the root zone via buried probes.	Large diameter root profiles, soil water profiles, etc.	Pros: Well-suited for dry soil (electrically resistive environments); act as a calibration	[151]
			Cons: Limited by the number of probe arrays that can be placed in the field; low throughput; time-consuming (up to 1 h/array)	
EMI	EMI is based on the spatial soil electrical conductivity by inductive coupling.	Soil water profiles, root architecture, etc.	Pros: Quick (less than 3 min) and repeatable method	[152]
			Cons: Limited traits; related to soil moisture content	
X-ray CT	X-ray CT is based on the x-rays’ attenuation to create cross-sections and then reconstruct 3D imaging of roots.	Root system architecture, patterning of lateral roots, root development, soil biota, etc.	Pros: Without requiring specific soils	[119]
			Cons: Time-consuming, pay attention to safety issues	
MRI	MRI is one imaging technique that employs radio-frequency waves and strong magnetic fields to stimulate atoms and produce 3D internal spatial information.	Root system architecture, root mass, length, diameter, tip number, growth angles, etc.	Pros: Noninvasive and can test in the field	[26]
			Cons: High-cost and time-consuming, relying on the soil condition	
PET	PET is based on detecting γ-rays from tracer molecules and visualizes the distribution of short half-life radioactive tracers, thus providing internal functioning information.	Root transportation function, root system architecture, length, diameter, growth angles, etc.	Pros: Noninvasive; quantitative and dynamic functional imaging of plants in 3D	[153]
			Cons: Time-consuming, low-throughput, relay on the soil condition, limited to a relatively coarse resolution	
MRI-PET	The combining technique of MRI and PET provides functional and structural 3D imaging.	Root system architecture, the dynamic changes in plant functions and structures, etc.	Pros: Measure the transportation statute and distribution of certain chemicals assimilated in plants	[118]
			Cons: High-cost, time-consuming, low-throughput, relay on the soil condition, limited to a relatively coarse resolution	

ERT, electrical resistance tomography; EMI, electromagnetic inductance.

## Compound Abiotic Stress Phenotyping

Commonly, plants would be exposed to several stressors simultaneously or subsequently in either the field or greenhouse, and their response to one individual stressor differs from the response to multiple stressors. Plant’s response to stress is markedly influenced by the stressor’s intensity, duration, and inherent factors of the plant, such as its species and growth stage. Additionally, it is essential to recognize that one stressor can trigger a chain reaction of other stressors, known as chain reaction stress, making it challenging to distinguish or identify complex or compound stress in plants.

**Fig. 3.**
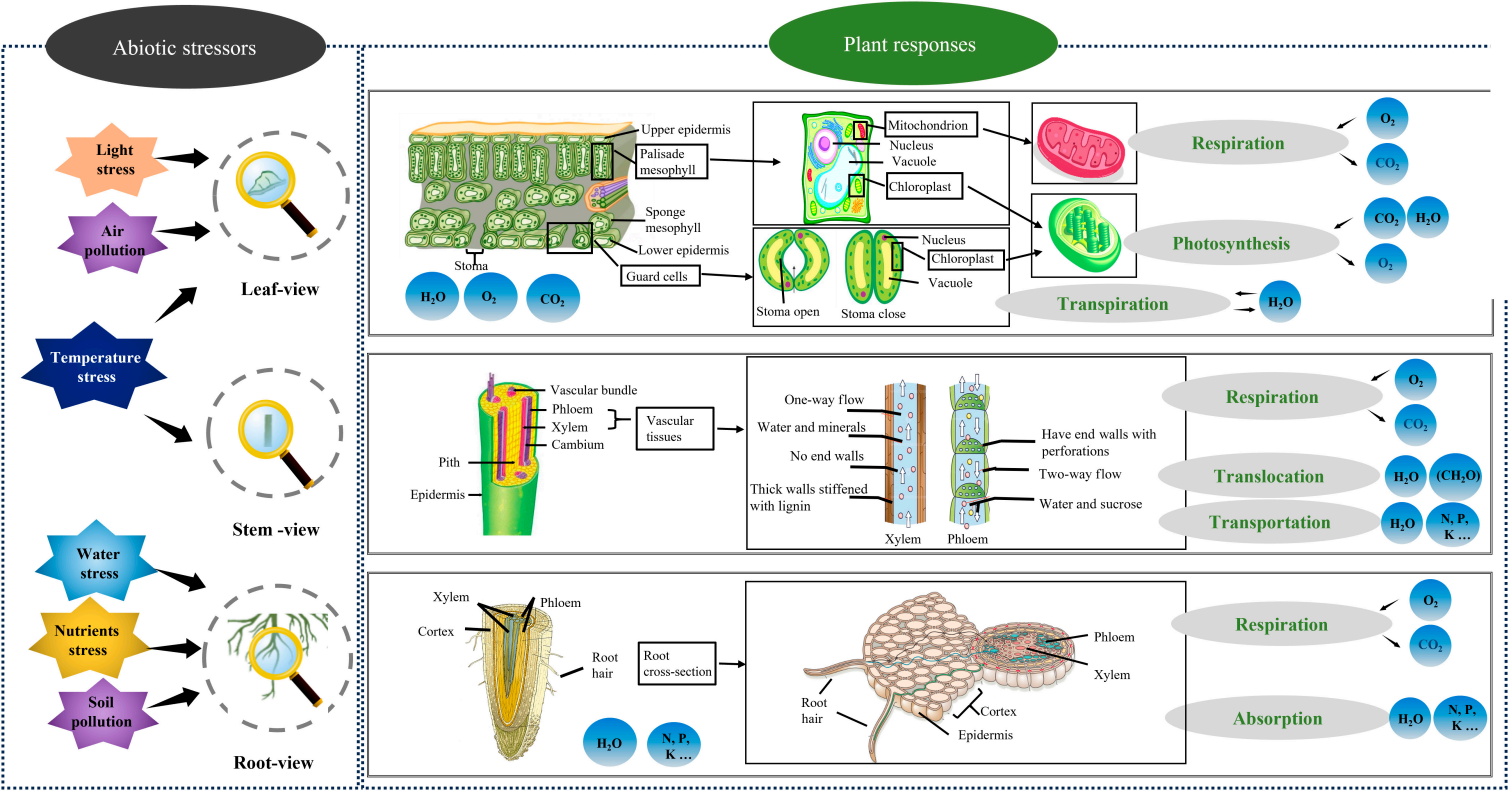
Plant perception and response to abiotic stress: insights into leaf, stem, and root reactions, mechanisms from macro to micro scales, main reaction organelles, and affected physiological functions.

However, considering the fact that plant response to stress begins by perceiving the stressor by a certain organ and then spreads to the whole plant, we can analyze the compound stress separately, and then assess the stress comprehensively. According to their location, environmental stressors are often first perceived by corresponding organs, such as the principle of proximity, and then influence their physiological activity and phenotypic information. This concept is illustrated in Fig. 3, which illustrates the process of plant perception and response to abiotic stress. It includes the mechanisms of corresponding organ responses from the macro to micro scale, the primary reaction organelles, and the main affected physiological functions. Thus, at the early stage of stress, the primary physiology activities that are affected include the following: leaf (photosynthesis + transpiration), stem (transportation + translocation), and root (absorption), as respiration, more precisely cellular respiration, is shared by the whole plant [39]. Meanwhile, there are also other physiological perturbations, such as membrane permeability and metabolism. In short, in leaf view, leaf perceives light stress and air pollution, affecting their physiological function (photosynthesis, transpiration, and respiration); in stem view, stem vascular tissues' function of water and nutrient transport is susceptible to cold stress; and in root view, root perceives water stress, nutrient stress, and soil pollution stress first.

The relationship between stressors could also be synthetically studied and described. For instance, Higley et al. [120] put forward the notion of stress interaction mode, which means identifying whether different stresses would be affected by one another. Hence, plant stress interaction can be classified into 2 main types: (a) Stress no-interaction mode: plant response to each stressor is independent of the occurrence of another, or failure to identify the relationship between them; (b) Stress interaction mode: plant response to one stressor is affected by the occurrence of others. Based on this, stress interaction mode can further be divided into 2 subtypes: stress exacerbation and stress alleviation. Stress exacerbation means the existence of one stressor can have an enhancing effect on the susceptibility to another stressor. In contrast, stress alleviation means that the presence of one stressor can alleviate the negative effect of another. The latter is also known as cross-adaption. The former case is the more common, where the exposure of plants to stress in combination can heighten the damage. For instance, heat stress could lead to increased transpiration, which could enhance salt uptake, exacerbating the salt-alkaline stress damage [121]. Stress alleviation or cross-adaption methods are also widely used for hardening seedlings. This effect may partly be due to the fact that specific stress accumulates the same general stress-response proteins; thus, plants can adjust more quickly.

**Fig. 4.**
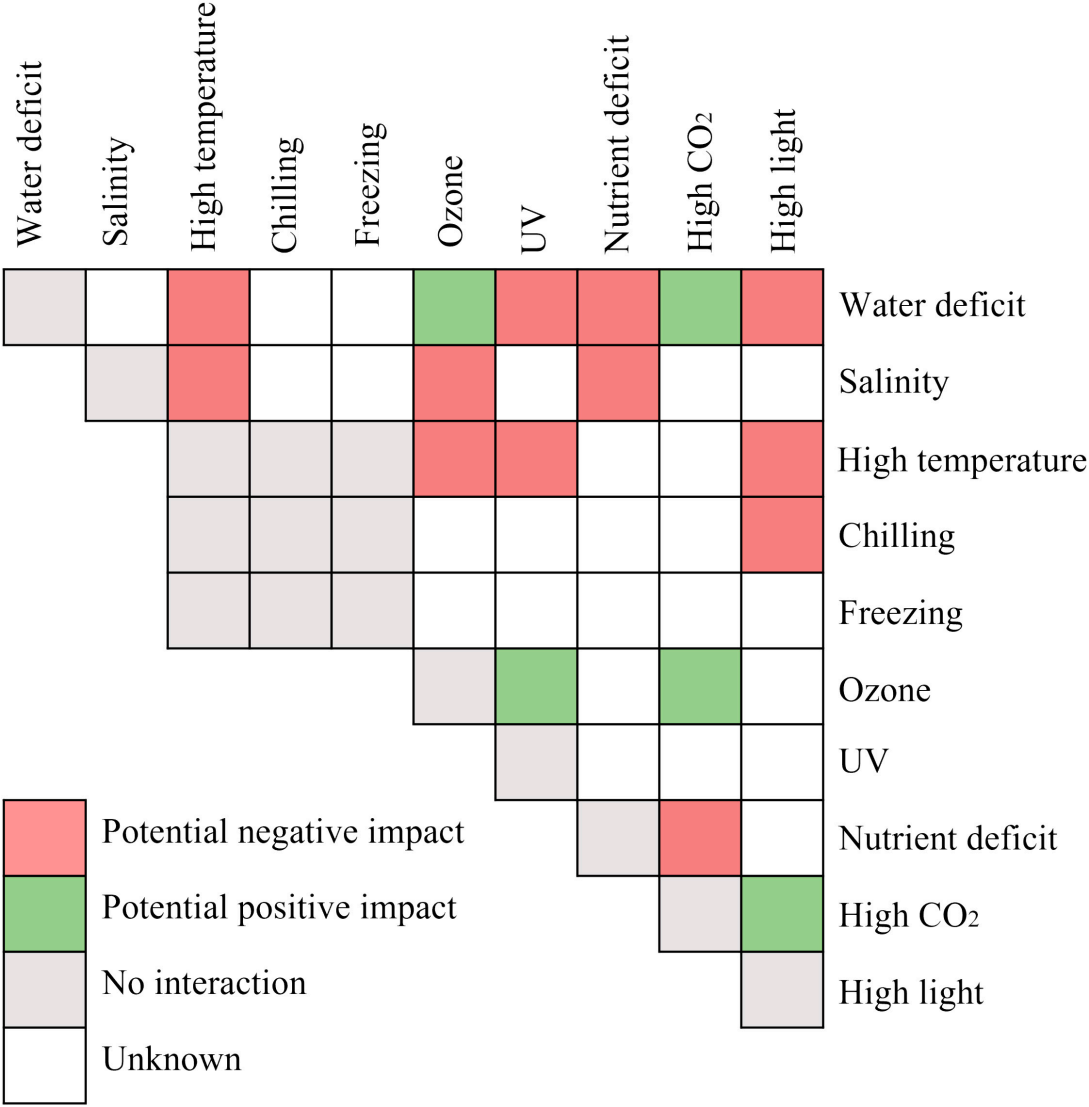
The compound abiotic stress interaction matrix. Colors indicate different stress combination effects.

The stress interaction modes of different environmental stress combinations are listed and shown as a stress matrix [39,122] in Fig. 4, from which we can indicate the stress interaction modes of different abiotic stressor combinations. To gain insights and distinguish complex or compound abiotic stress, we delve into the response processes of different organs, assessing their interactions. This synthesis analysis aids in diagnosing compound stress, particularly in distinguishing multiple abiotic stressors and identifying the key tissue responses required to overcome the stress.

## Conclusions and Perspectives

In this paper, noninvasive phenotyping technologies related to plant abiotic stress have been systematically reviewed, along with the physiological reactions and phenotypic information of each vegetative organ under stress. These studies can provide early warning signals and help distinguish between stress types. The novel insights of the multi-organ, noninvasive phenotyping study provide a reference for testing hypotheses concerning the intricate dynamics of plant stress responses, as well as the potential interactive effects among various stressors. Currently, the leaf view is the mainstream, the root view is crucial for peering at the underground part, and others (fruit, flower, and seed) are mainly for propagation or yield estimation. In contrast, the stem view is often the choice being left out, either for subjective (ignored) or objective (opaque) reasons. Different phenotyping techniques specialize in measuring distinct indices. Therefore, combining available techniques for a comprehensive analysis of each organ’s response to compound and complex abiotic stress is an alternative approach. In summary, noninvasive yet precise phenotyping is essential for advancing phenotypic plasticity research and can prompt delving into the molecular mechanisms of gene expression under abiotic stress. Although it remains relatively understudied due to the limitations of available techniques, the potential rewards it holds for improving plant well-being are highly promising.

## Data Availability

Data are availablefrom theauthors upon reasonable request.
